# Complete Mitochondrial Genomes Reveal Neolithic Expansion into Europe

**DOI:** 10.1371/journal.pone.0032473

**Published:** 2012-03-13

**Authors:** Qiaomei Fu, Pavao Rudan, Svante Pääbo, Johannes Krause

**Affiliations:** 1 Department of Evolutionary Genetics, Max Planck Institute for Evolutionary Anthropology, Leipzig, Germany; 2 Department of Natural Sciences, Croatian Academy of Sciences and Arts, Zagreb, Croatia; 3 Institute for Archaeological Sciences, University of Tübingen, Tübingen, Germany; Institut de Biologia Evolutiva - Universitat Pompeu Fabra, Spain

## Abstract

The Neolithic transition from hunting and gathering to farming and cattle breeding marks one of the most drastic cultural changes in European prehistory. Short stretches of ancient mitochondrial DNA (mtDNA) from skeletons of pre-Neolithic hunter-gatherers as well as early Neolithic farmers support the demic diffusion model where a migration of early farmers from the Near East and a replacement of pre-Neolithic hunter-gatherers are largely responsible for cultural innovation and changes in subsistence strategies during the Neolithic revolution in Europe. In order to test if a signal of population expansion is still present in modern European mitochondrial DNA, we analyzed a comprehensive dataset of 1,151 complete mtDNAs from present-day Europeans. Relying upon ancient DNA data from previous investigations, we identified mtDNA haplogroups that are typical for early farmers and hunter-gatherers, namely H and U respectively. Bayesian skyline coalescence estimates were then used on subsets of complete mtDNAs from modern populations to look for signals of past population expansions. Our analyses revealed a population expansion between 15,000 and 10,000 years before present (YBP) in mtDNAs typical for hunters and gatherers, with a decline between 10,000 and 5,000 YBP. These corresponded to an analogous population increase approximately 9,000 YBP for mtDNAs typical of early farmers. The observed changes over time suggest that the spread of agriculture in Europe involved the expansion of farming populations into Europe followed by the eventual assimilation of resident hunter-gatherers. Our data show that contemporary mtDNA datasets can be used to study ancient population history if only limited ancient genetic data is available.

## Introduction

Archaeological evidence suggests that agrarian societies emerged in Western Asia around 11,000 years before present (YBP) [1] and rapidly spread reaching South Eastern Europe by approximately 9,000 YBP [2]. The transition from pre-Neolithic hunter-gatherer societies to Neolithic farming and cattle breeding is often called the Neolithic revolution and marks one of the most pronounced cultural changes in European prehistory [3], [4] that can be observed in the archaeological record all over Europe [5]. By around 5,000 YBP almost all populations in mainland Europe practiced agriculture. There are two main hypotheses for how Neolithic cultures spread across Europe. The first, suggests cultural transmission as the main factor, i.e. that the new technologies and subsistence strategies were learned from neighbouring groups [6]. The second hypothesis suggests an expansion of farmer populations from the Near East into Europe, replacing most of the pre-Neolithic hunter-gatherer populations. This population replacement model, termed demic diffusion, is conceived as population spread and expansion, with limited admixture with resident populations.

**Figure 1:**
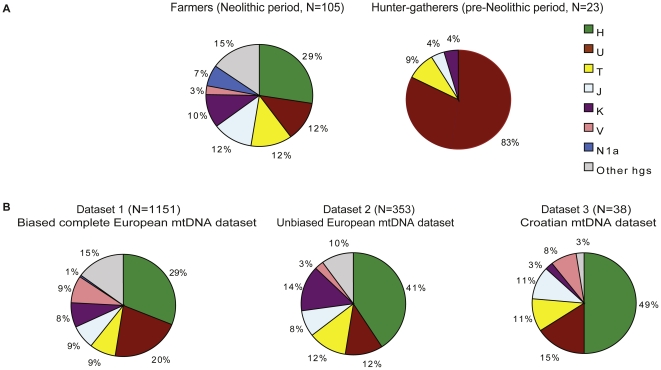
Haplogroup frequencies of hunter-gatherer and early farmer populations based on short segments of the mtDNA (A); Haplogroup frequencies of three contemporary European complete mtDNA datasets (B).

Recently, mitochondrial DNA (mtDNA) from skeletal remains of European early farmers and late hunter-gatherers has been retrieved [7]–[13]. The frequency of mtDNA haplogroups, defined by substitutions shared by related mtDNA types (Phylotree.org-mtDNA tree build 12), in early farmers across Europe [7], [10]–[13] was found to be overall similar to those in modern Europeans (Figure 1, Figure S4, Figure S5), while pre-Neolithic hunter-gatherers appear to be quite distinct (Figure 1). In particular, 83% (19 out of 23) of hunter-gatherers analyzed to date carry mtDNAs belonging to haplogroup U [9], [10], [14] and none of the hunter-gatherers fall in haplogroup H. In contrast, haplogroup U has been found in only 13 of 105 (around 12%) individuals from early farming cultures of Europe and it occurs in less than 21% of modern Europeans, while haplogroup H comprises between 25% and 37% of mtDNAs retrieved from early farming cultures (Figure S4) and is in about 30% of contemporary Europeans (Figure 1). The mtDNA data thus suggest that the pre-Neolithic populations in Europe were largely replaced by in-coming Neolithic farming groups, with a maximum mtDNA contribution of around 20% from pre-Neolithic hunter-gatherers [8]–[10]. The genetic contribution of pre-Neolithic hunter-gatherers to later Neolithic populations is furthermore supported by a similar frequency of U subhaplogroups (U5, U4, K and U2) that were found in pre-Neolithic hunter-gatherers (Figure S3) and are still the most common U-subhaplogroups in modern Central Europeans (Figure S5).

The mtDNA sequences determined from early farmers and hunter-gatherers are however less than 400 bp in length and their number is quite small (105 and 21, respectively), limiting the information that can be gained about population sizes and putative population expansions in the past. Here, we use a total of 1,151 complete mtDNAs from present-day populations in Europe, along with 38 mtDNAs which we determined from a modern population in Croatia, to estimate the frequency of the haplogroup U, putatively typical of hunter-gatherers, and mtDNAs of the haplogroup H, putatively typical of the early farming cultures. We then use these data to study potential differences in signatures of demographic history of hunter-gatherers and farmers in Europe that are discernable in present-day European mtDNAs.

## Results and Discussion

A total of 1,151 complete mtDNA sequences from present-day Europeans were collected from GenBank (dataset 1). Due to various ascertainment biases, such as selected sequencing of rare variants [15]–[18] in this data set, which might influence the analysis and conclusions drawn, we first used an unbiased randomly selected subset of 259 complete mtDNAs from all of Europe (dataset 2) [19]. Secondly, to test for potential non-reported ascertainment biases in dataset 2, we furthermore generated 38 complete mtDNAs from random villagers from Croatia (dataset 3). In each data set, mtDNAs of the U-type and H-type were identified (Table 1, Table 2, Table 3).

**Table 1:** Geographic origin, number and haplogroup designation for complete European mtDNA dataset.

Country	Continent	Number	Haplogroup	Source
Finland	Europe	31	H	[33]
Italy	Europe	119	H	[17], [34]–[37]
Poland	Europe	2	H	[18]
Portugal	Europe	17	H	[16], [38]
Slovakia	Europe	3	H	[18]
Spain	Europe	8	H	[37], [39], [40]
Basque	Europe	8	H	[41]
**Not specified**	Europe	144	H	[19]
Belarus	Europe	6	U	[18]
Bosnia and Herzegovina	Europe	1	U	[15]
Bulgaria	Europe	1	U	[15]
Croatia	Europe	1	U	[15]
Czech Republic	Europe	7	U	[18]
Estonia	Europe	1	U	[15]
Finland	Europe	31	U	[33]
France	Europe	3	U	[15], [17]
Germany	Europe	2	U	[15]
Greece	Europe	1	U	[15]
Hungary	Europe	1	U	[42]
Italy	Europe	74	U	[15], [17], [35], [36], [43], [44]
Poland	Europe	19	U	[18]
Portugal	Europe	3	U	[16]
Scotland	Europe	1	U	[15]
Slovakia	Europe	9	U	[18]
Spain	Europe	23	U	[15]
Sami	Europe	3	U	[44]
**Not specified**	Europe	41	U	[19]

**Table 2:** Number and haplogroup designation of complete mtDNA from a non- biased source [19].

Country	Continent	Number	Haplogroup	Source
**Not specified**	Europe	144	H	[19]
**Not specified**	Europe	41	U	[19]

**Table 3:** Number and haplogroup designation of complete mtDNAs from two Croatian villages.

Country	Continent	Number	Haplogroup	Source
Croatia	Europe	19	H	GenBank accession numbers in Table S1
Croatia	Europe	6	U	GenBank accession numbers in Table S1

**Figure 2:**
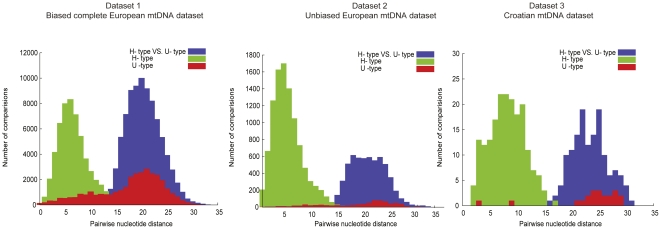
Pairwise nucleotide differences for all U- type and H- type mtDNAs in three contemporary European datasets.

Whereas H-type mtDNAs have on average six nucleotide differences in their coding region (position 577–16023) (Figure 2, green), U-type mtDNAs have on average 18 differences (Figure 2, red). The distribution of pair-wise differences among the H-type mtDNAs shows a clear mode around 6 differences whereas the U-types have a mode around 22 differences. Such peaks may be caused by past population expansions [20] (Figure S7, Figure S8, Figure S9). They would suggest that H-type mtDNAs experienced a recent population expansion while U-type mtDNAs experienced a much older population expansion. Notably, these differences in the distributions of pair-wise nucleotide differences are not caused by sequencing of a selected set of mtDNA types present in GenBank, since dataset 2 as well as the individuals sequenced from Croatia (dataset 3) show an average number of differences as well as modes very similar to dataset 1.

**Figure 3:**
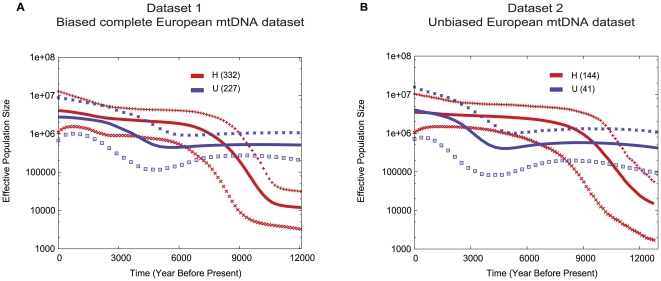
Estimated effective population size (Ne) of type H (red) and type U mtDNA haplotypes (blue) for the complete European mtDNA dataset as well as for the sampled dataset. The x-axis shows time in years before present, the y axis the effective population size Ne. The center line represents the mean of Ne estimate, upper and lower lines are the 95% posterior density intervals. We assumed a mutation rate of the coding regions of 1.691×10^−8^ substitutions per site and year [30]–[32].

In order to analyze potential population size changes over time, we calculated Bayesian skyline plots using the BEAST package [21] for dataset 1 and dataset 2 (dataset 3 was too small). In both datasets, the direct comparison of skyline plots between the H-type and the U-type mtDNAs (Figure 3) reveals a population increase for individuals carrying the H-type starting around 9,000 YBP and continuing to the present, whereas the U-type shows a population expansion between 20,000 and 10,000 YBP with a putative period of slight decrease between 6,000 and 5,000 YBP (Figure S6A, B). For both U-type and H-type mtDNAs, we observe similar patterns of population growth starting around 4,000 YBP to the present (Figure 3). Thus, H-type and U-type mtDNAs show a distinct population history before 5,000 YBP, possibly reflecting that they were primarily present in different populations with different origins and histories.

The high frequency of H-type mtDNAs in European Neolithic populations and its complete absence in pre-Neolithic hunter-gatherers suggests that H-type mtDNAs arrived with early farmers in Europe. The population size increase observed between 9,000 and 5,000 YBP likely represents the population expansion that accompanied the Neolithic revolution. In contrast, U-type mtDNAs show an increase in population size around 15,000 to 10,000 YBP, which coincides with the end of the last glacial maximum in Europe and a northwards expansion of hunter-gatherer populations. The data suggests that this population remained rather constant after 10,000 YBP until the onset of the Neolithic revolution. However, the H-type mtDNA population size seems to experience an exponential increase around 7,000 YBP, suggesting that both populations are not yet fused. After 4,000 YBP, no archaeological remains of hunter-gatherers were found in central Europe [22]. From approximately that time on, both H- and U-type mtDNAs expand in a similar way. This may reflect fusion of the two populations where these mtDNAs were prevalent.

These results suggest that H-type mtDNAs in the European mtDNA gene pool show evidence of a population expansion related to the spread of animal husbandry and farming. In contrast, U-type mtDNAs seem to represent earlier hunter-gatherers that adopted farming practices and admixed with immigrant farming populations. In agreement with this scenario, the only non-agricultural population of Europe, the Saami in Northern Scandinavia and Russia, carry about 49% of U-type mtDNAs [23].

## Materials and Methods

### DNA Sequence Data

Due to the high mutation rate and the risk of homoplasy, we excluded non-coding regions from our analysis. We identified haplogroups for each mtDNA using the database phylotree (based on Phylotree.org-mtDNA build 12). For the whole European mtDNA dataset comprising 1,151 sequences we identified 332 mtDNAs falling into haplogroup H, representing farmers for our purposes, and 227 mtDNAs falling into haplogroup U, typical for early hunter-gatherers (Figure 1B). For the sampled 259 population-wide data, we identified 144 mtDNAs of type H and 41 of type U. Further, we enriched, sequenced and assembled mitochondrial genomes (Supporting Method S1) from a contemporary populations of villagers sampled in the Northeast and Northwest of Croatia (Figure S1, Figure S2, Table S1). In this Croatian dataset we identified 19 mtDNA sequences of type H and 6 of type U (Figure 1B).

### Evolutionary Analysis

Pairwise nucleotide distances were calculated using MEGA 4 [24]. Skyline plots were estimated using coding regions (positions 577–16023) from the U- and the H- type mtDNA datasets using the Bayesian algorithm of BEAST v1.5.3 [25]. The General Time Reversible sequence evolution model with a fixed fraction of invariable sites (GTR+I) was determined by the best-fit model approach of Modeltest and PAUP* [26]. For each analysis, we used parallel models that assumes a Bayesian skyline coalescent and a constant size coalescent across the phylogeny and ran 50,000,000 generations of the Markov Chain Monte Carlo with the first 5,000,000 generations discarded as burn-in. Final model was chosen by using Bayes factors (BF>20 is strong support for the favored model [27]–[29], and reported as log_10_ Bayes factors (log_10_ BF). Here the Bayesian skyline model fits the data better than constant population size in H-type (dataset 1: log_10_ = 2.69; dataset 2 log_10_ = 6.86). And the Bayesian skyline model cannot be rejected in U-type (dataset 1: log_10_ = 0.34; dataset 2 log_10_ = 0.91). The alignment was analyzed using a strict molecular clock with a substitution rate of 1.691×10^−8^ substitutions per site and year [30]–[32].

## Supporting Information

Figure S1 **Map of villages sampled in the Northeast and Northwest of Croatia.**
(TIF)

Figure S2 **Read coverage (logarithmic scale; upper part) and GC content (lower part) along the complete mitochondrial genome for the 50 Croatian samples.** Coverage is not highly correlated with GC content.
(TIF)

Figure S3 **Haplogroup frequency of pre-Neolithic samples.**
(TIF)

Figure S4 **Haplogroup frequency of Neolithic samples.**
(TIF)

Figure S5 **Haplogroup frequency of modern human sets.**
(TIF)

Figure S6 **Estimates for the effective population size (Ne) over time for haplotype U mtDNA sequences for the full (A) and the subsampled (B) European mtDNA datasets over 35,000 years.** Estimated effective population size (Ne) over time of type H (red) and type U5 mtDNA haplotypes (blue) for the complete European mtDNA dataset (C) as well as for the sampled dataset (D). Estimates for the effective population size (Ne) for haplotype U5 mtDNA sequences for the full (E) and the sampled (F) European mtDNA datasets over 16,000 years. The x-axis shows time in years before present, the y axis the effective population size Ne. The center line represents the mean of Ne estimate, upper and lower lines are the 95% posterior density intervals. We assumed a mutation rate of the coding regions of 1.691×10^−8^ substitutions per site and year.
(TIF)

Figure S7 **Phylogenetic tree of mtDNAs from dataset 1.** The phylogeny was estimated with a Bayesian approach under a GTR+I+R model using 332 present-day European mtDNA sequences of haplogroup H and 228 sequences from haplogroup U. The outgroup is a African mtDNA sequence.
(TIF)

Figure S8 **Phylogenetic tree of mtDNAs from dataset 2.** The phylogeny was estimated with a Bayesian approach under a GTR+I+R model using 144 present-day European mtDNA sequences of haplogroup H and 41 sequences from haplogroup U. The outgroup is the African mtDNA sequence.
(TIF)

Figure S9 **Phylogenetic tree of mtDNAs of dataset 3.** The phylogeny was estimated with a Bayesian approach under a GTR+I+R model using 20 present-day Croatian mtDNA sequences of haplogroup H and 7 sequences from haplogroup U. The outgroup is the African mtDNA sequence.
(TIF)

Supporting Method S1 **Supporting sequence information of Croatians.**
(DOC)

Table S1 **Sequence information of 50 Croatian mtDNA sequences.** Samples in italic were removed from further analysis.
(XLS)

## References

[1] Daniel Zohary MH (1993). Domestication of Plants in the Old World: The Origin and Spread of Cultivated Plants in West Asia, Europe, and the Nile Valley.

[2] Greenfield H, Robertson JDS ElizabethC, Fernandez DeepikaC, Zender MarcU (2006). The spatial organization of Early Neolithic settlements in temperate southeastern Europe: a view from Blagotin, Serbia.. In Space and Spatial Analysis in Archaeology.

[3] Alasdair Whittle VC (2007). Going over: the mesolithic-neolithic transition in North-West Europe.

[4] Zvelebil M (1989). On the transition to farming in Europe, or what was spreading with the Neolithic: a relay to Ammerman.. Antiqutiy.

[5] Harris DR (1996). The Origins and Spread of Agriculture and Pastoralism in Eurasia.

[6] Alena Lukes MZ (2004). LBK Dialogues: Studies in the Formation of the Linear Pottery Culture.

[7] Sampietro ML, Lao O, Caramelli D, Lari M, Pou R (2007). Palaeogenetic evidence supports a dual model of Neolithic spreading into Europe.. Proceedings of the Royal Society B-Biological Sciences.

[8] Haak W, Forster P, Bramanti B, Matsumura S, Brandt G (2005). Ancient DNA from the first European farmers in 7500-year-old Neolithic sites.. Science.

[9] Bramanti B, Thomas MG, Haak W, Unterlaender M, Jores P (2009). Genetic Discontinuity Between Local Hunter-Gatherers and Central Europe's First Farmers.. Science.

[10] Haak W, Balanovsky O, Sanchez JJ, Koshel S, Zaporozhchenko V (2010). Ancient DNA from European early neolithic farmers reveals their near eastern affinities.. PLoS Biol.

[11] Deguilloux MF, Soler L, Pemonge MH, Scarre C, Joussaume R (2011). News From the West: Ancient DNA From a French Megalithic Burial Chamber.. American Journal of Physical Anthropology.

[12] Lacan M, Keyser C, Ricaut FX, Brucato N, Duranthon F (2011). Ancient DNA reveals male diffusion through the Neolithic Mediterranean route.. Proceedings of the National Academy of Sciences of the United States of America.

[13] Gamba C, Fernandez E, Tirado M, Deguilloux MF, Pemonge MH (2012). Ancient DNA from an Early Neolithic Iberian population supports a pioneer colonization by first farmers.. Mol Ecol.

[14] Krause J, Briggs AW, Kircher M, Maricic T, Zwyns N (2010). A complete mtDNA genome of an early modern human from Kostenki, Russia.. Curr Biol.

[15] Pala M, Achilli A, Olivieri A, Kashani BH, Perego UA (2009). Mitochondrial haplogroup U5b3: a distant echo of the epipaleolithic in Italy and the legacy of the early Sardinians.. American Journal of Human Genetics.

[16] Pereira L, Goncalves J, Franco-Duarte R, Silva J, Rocha T (2007). No evidence for an mtDNA role in sperm motility: Data from complete sequencing of asthenozoospermic males.. Molecular Biology and Evolution.

[17] Carelli V, Achilli A, Valentino ML, Rengo C, Semino O (2006). Haplogroup effects and recombination of mitochondrial DNA: Novel clues from the analysis of Leber hereditary optic neuropathy pedigrees.. American Journal of Human Genetics.

[18] Malyarchuk B, Grzybowski T, Derenko M, Perkova M, Vanecek T (2008). Mitochondrial DNA Phylogeny in Eastern and Western Slavs.. Molecular Biology and Evolution.

[19] Herrnstadt C, Elson JL, Fahy E, Preston G, Turnbull DM (2002). Reduced-median-network analysis of complete mitochondrial DNA coding-region sequences for the major African, Asian, and European haplogroups.. American Journal of Human Genetics.

[20] Rogers AR, Harpending H (1992). Population growth makes waves in the distribution of pairwise genetic differences.. Molecular Biology and Evolution.

[21] Atkinson QD, Gray RD, Drummond AJ (2008). mtDNA Variation Predicts Population Size in Humans and Reveals a Major Southern Asian Chapter in Human Prehistory.. Molecular Biology and Evolution.

[22] Nowark M (2007). Middle and Late Holocene hunter-gatherers in East Central Europe: changing paradigms of the ‘non-Neolithic’ way of life.

[23] Tambets K, Rootsi S, Kivisild T, Help H, Serk P (2004). The Western and Eastern Roots of the Saami–the Story of Genetic “Outliers” Told by Mitochondrial DNA and Y Chromosomes.. The American Journal of Human Genetics.

[24] Tamura K, Dudley J, Nei M, Kumar S (2007). MEGA4: Molecular Evolutionary Genetics Analysis (MEGA) software version 4.0.. Molecular Biology and Evolution.

[25] Drummond A, Rambaut A (2007). BEAST: Bayesian evolutionary analysis by sampling trees.. BMC Evolutionary Biology.

[26] Posada D, Crandall KA (1998). MODELTEST: testing the model of DNA substitution.. Bioinformatics.

[27] Newton MA, Raftery AE, Davison AC, Bacha M, Celeux G (1994). Approximate Bayesian-Inference with the Weighted Likelihood Bootstrap.. Journal of the Royal Statistical Society Series B-Methodological.

[28] Drummond AJ, Rambaut A (2007). BEAST: Bayesian evolutionary analysis by sampling trees.. BMC Evol Biol.

[29] Suchard MA, Weiss RE, Sinsheimer JS (2001). Bayesian selection of continuous-time Markov chain evolutionary models.. Molecular Biology and Evolution.

[30] Ho SY, Phillips MJ, Cooper A, Drummond AJ (2005). Time dependency of molecular rate estimates and systematic overestimation of recent divergence times.. Molecular Biology and Evolution.

[31] Friedlaender J, Schurr T, Gentz F, Koki G, Friedlaender F (2005). Expanding Southwest Pacific mitochondrial haplogroups P and Q.. Molecular Biology and Evolution.

[32] Schonberg A, Theunert C, Li M, Stoneking M, Nasidze I (2011). High-throughput sequencing of complete human mtDNA genomes from the Caucasus and West Asia: high diversity and demographic inferences.. Eur J Hum Genet.

[33] Finnilä S, Lehtonen MS, Majamaa K (2001). Phylogenetic Network for European mtDNA.. The American Journal of Human Genetics.

[34] Pello R, Martin MA, Carelli V, Nijtmans LG, Achilli A (2008). Mitochondrial DNA background modulates the assembly kinetics of OXPHOS complexes in a cellular model of mitochondrial disease.. Hum Mol Genet.

[35] Gasparre G, Porcelli AM, Bonora E, Pennisi LF, Toller M (2007). Disruptive mitochondrial DNA mutations in complex I subunits are markers of oncocytic phenotype in thyroid tumors.. Proc Natl Acad Sci U S A.

[36] Fraumene C, Belle EMS, Castrì L, Sanna S, Mancosu G (2006). High Resolution Analysis and Phylogenetic Network Construction Using Complete mtDNA Sequences in Sardinian Genetic Isolates.. Molecular Biology and Evolution.

[37] Achilli A, Rengo C, Magri C, Battaglia V, Olivieri A (2004). The molecular dissection of mtDNA haplogroup H confirms that the Franco-Cantabrian glacial refuge was a major source for the European gene pool.. American Journal of Human Genetics.

[38] Behar DM, Metspalu E, Kivisild T, Rosset S, Tzur S (2008). Counting the founders: the matrilineal genetic ancestry of the Jewish Diaspora.. PLoS One.

[39] Ennafaa H, Cabrera VM, Abu-Amero KK, Gonzalez AM, Amor MB (2009). Mitochondrial DNA haplogroup H structure in North Africa.. BMC Genet.

[40] Maca-Meyer N, Gonzalez AM, Larruga JM, Flores C, Cabrera VM (2001). Major genomic mitochondrial lineages delineate early human expansions.. BMC Genet.

[41] Álvarez-Iglesias V, Mosquera-Miguel A, Cerezo M, Quintáns B, Zarrabeitia MT (2009). New Population and Phylogenetic Features of the Internal Variation within Mitochondrial DNA Macro-Haplogroup R0.. PLoS One.

[42] Maasz A, Komlosi K, Hadzsiev K, Szabo Z, Willems PJ (2008). Phenotypic variants of the deafness-associated mitochondrial DNA A7445G mutation.. Curr Med Chem.

[43] Brisighelli F, Capelli C, Alvarez-Iglesias V, Onofri V, Paoli G (2009). The Etruscan timeline: a recent Anatolian connection.. Eur J Hum Genet.

[44] Achilli A, Rengo C, Battaglia V, Pala M, Olivieri A (2005). Saami and Berbers An Unexpected Mitochondrial DNA Link.. American Journal of Human Genetics.

