# Supplementary figures and images for: Complete Mitochondrial Genomes Reveal Neolithic Expansion into Europe
Source: PLoS One. 2012 Mar 13;7(3):e32473. doi: 10.1371/journal.pone.0032473 (PMC3302788; doi:10.1371/journal.pone.0032473)

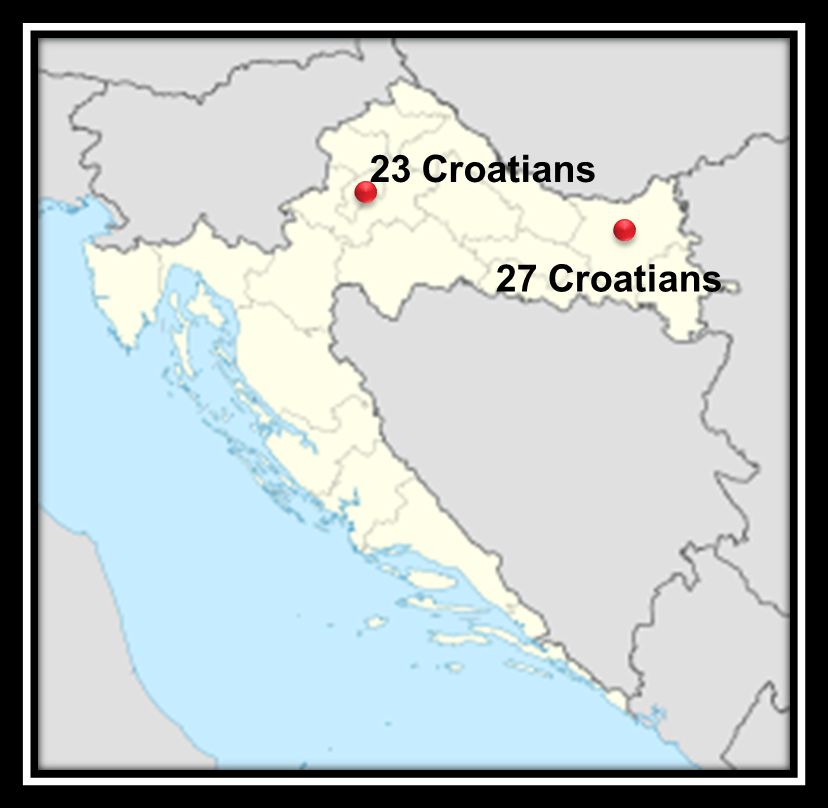

Supplement: Figure S1 — Map of villages sampled in the Northeast and Northwest of Croatia. (TIF) [file pone.0032473.s001.tif]

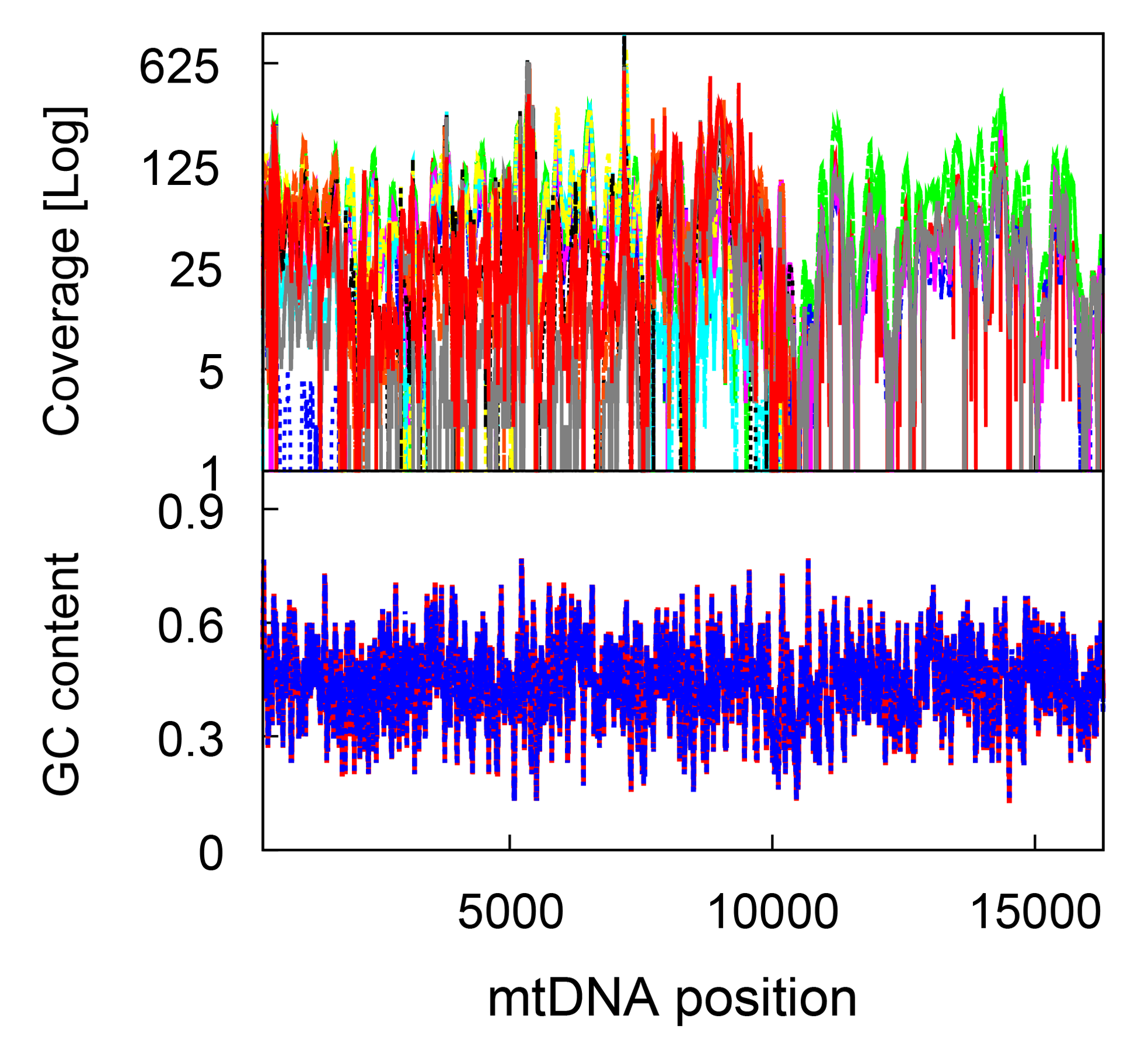

Supplement: Figure S2 — Read coverage (logarithmic scale; upper part) and GC content (lower part) along the complete mitochondrial genome for the 50 Croatian samples. Coverage is not highly correlated with GC content. (TIF) [file pone.0032473.s002.tif]

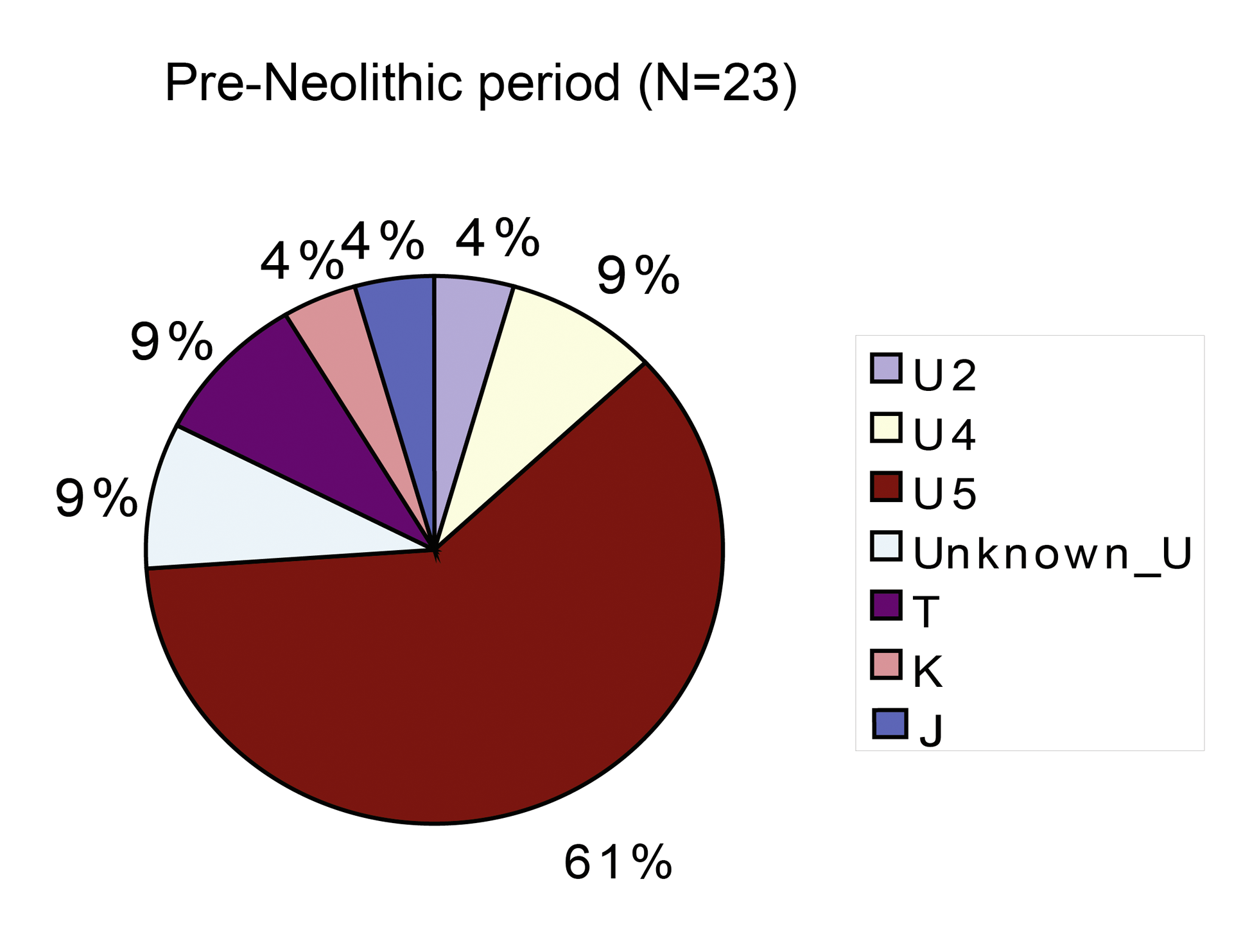

Supplement: Figure S3 — Haplogroup frequency of pre-Neolithic samples. (TIF) [file pone.0032473.s003.tif]

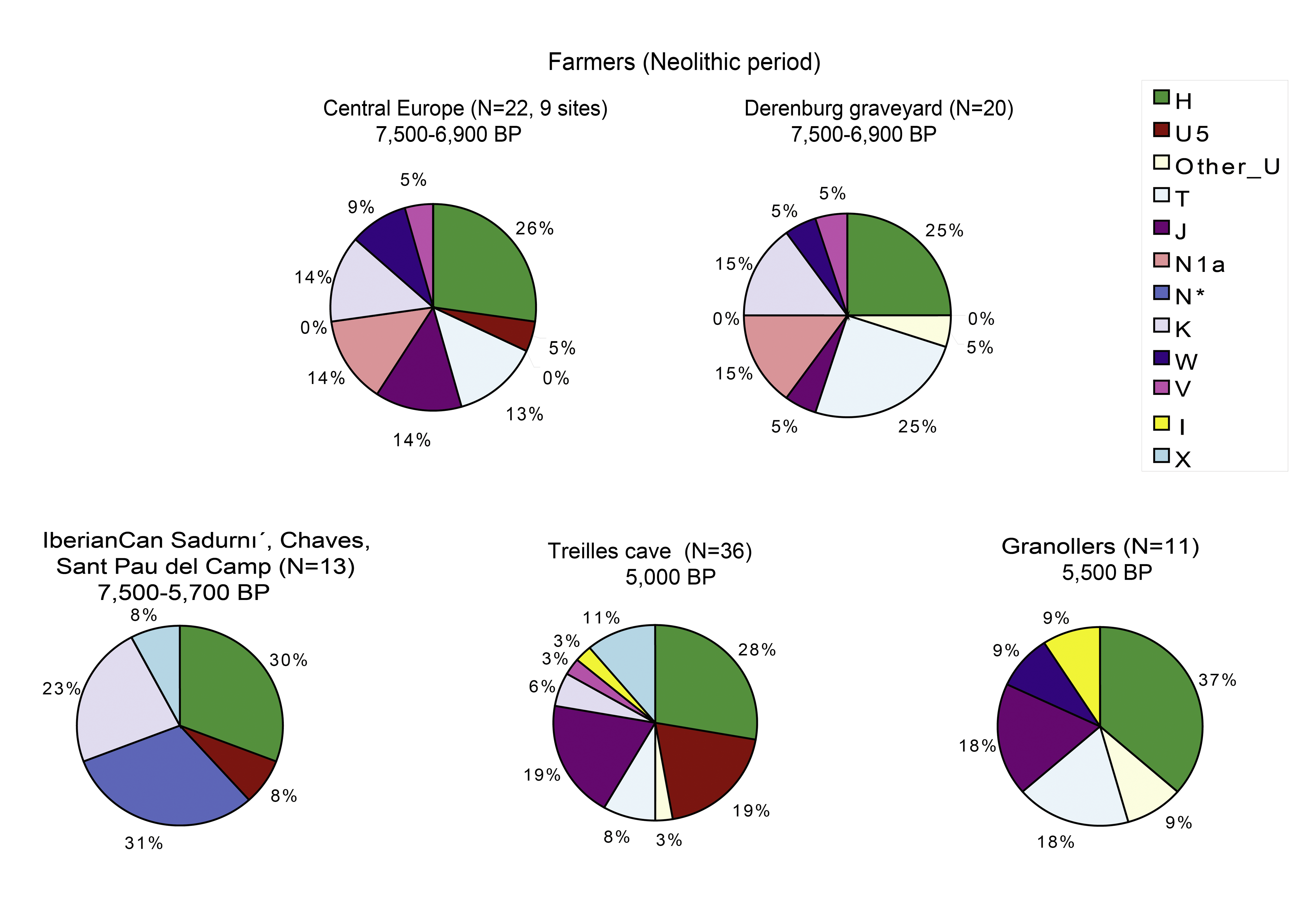

Supplement: Figure S4 — Haplogroup frequency of Neolithic samples. (TIF) [file pone.0032473.s004.tif]

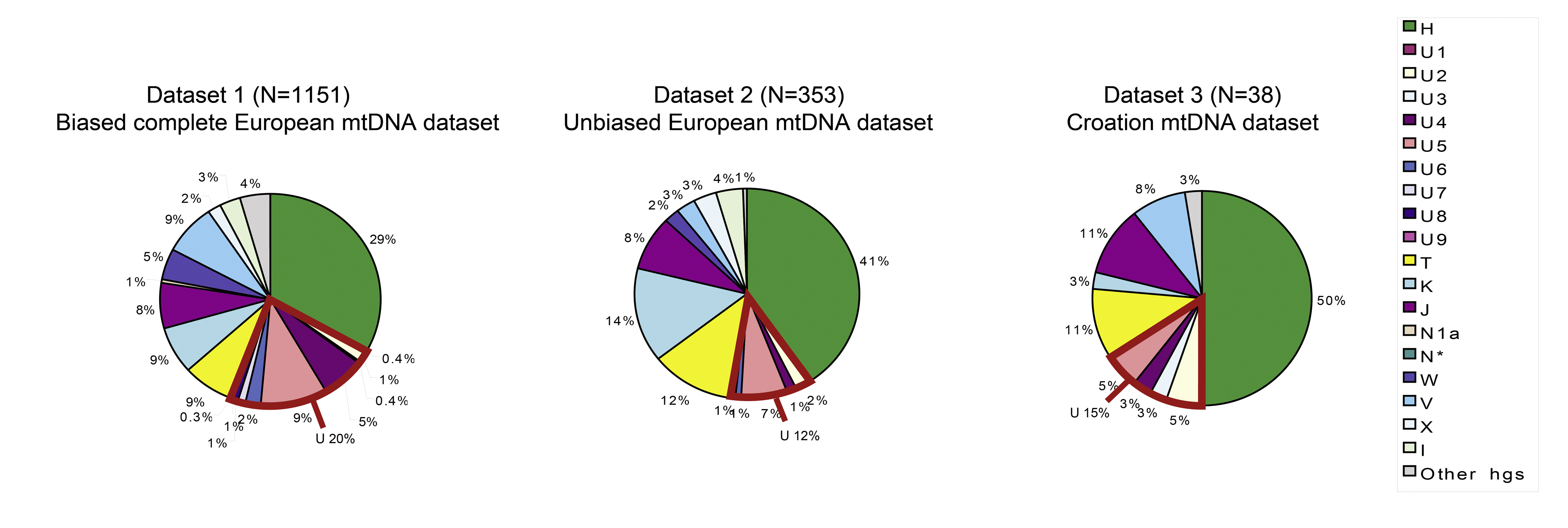

Supplement: Figure S5 — Haplogroup frequency of modern human sets. (TIF) [file pone.0032473.s005.tif]

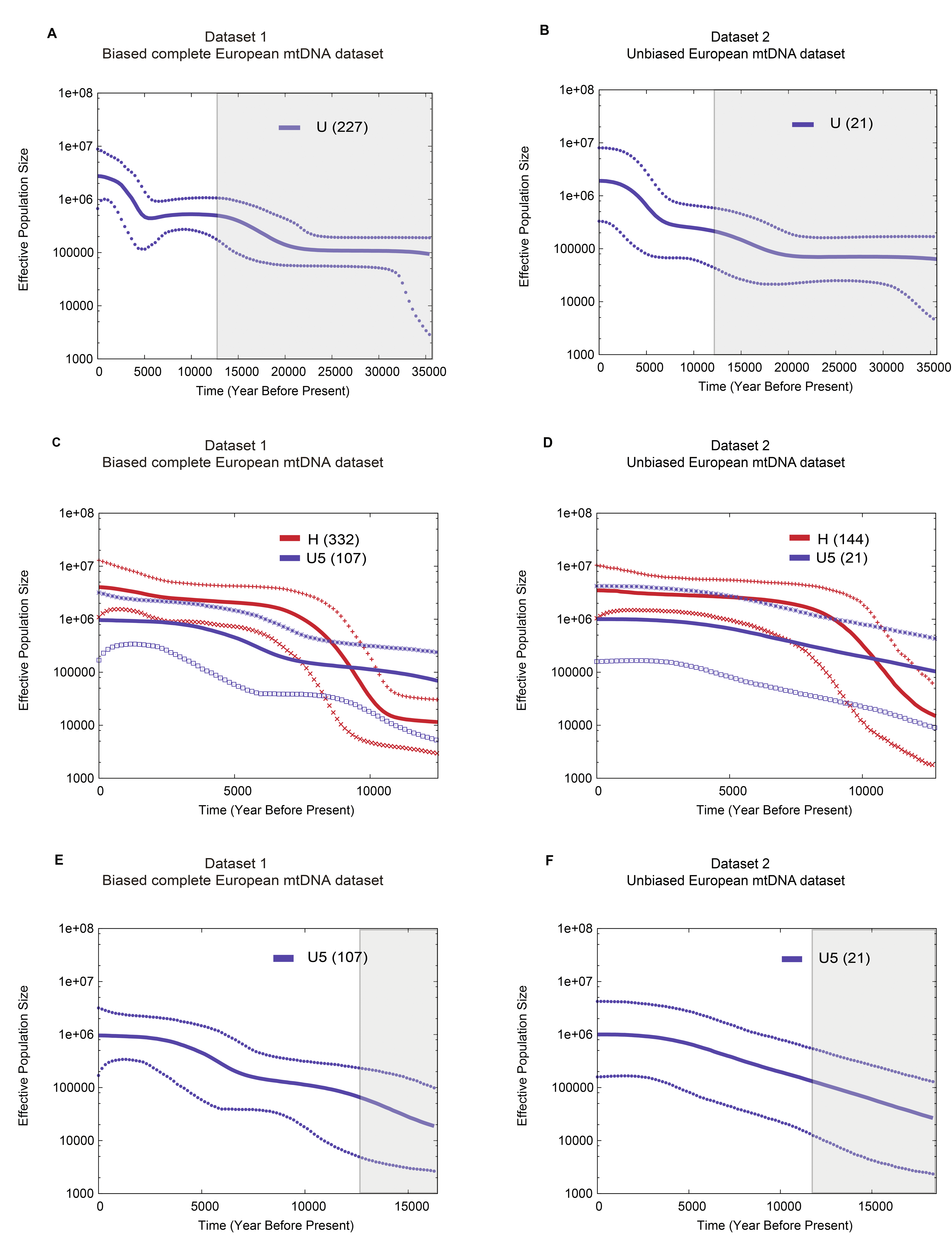

Supplement: Figure S6 — Estimates for the effective population size (Ne) over time for haplotype U mtDNA sequences for the full (A) and the subsampled (B) European mtDNA datasets over 35,000 years. Estimated effective population size (Ne) over time of type H (red) and type U5 mtDNA haplotypes (blue) for the complete European mtDNA dataset (C) as well as for the sampled dataset (D). Estimates for the effective population size (Ne) for haplotype U5 mtDNA sequences for the full (E) and the sampled (F) European mtDNA datasets over 16,000 years. The x-axis shows time in years before present, the y axis the effective population size Ne. The center line represents the mean of Ne estimate, upper and lower lines are the 95% posterior density intervals. We assumed a mutation rate of the coding regions of 1.691×10^−8^ substitutions per site and year. (TIF) [file pone.0032473.s006.tif]

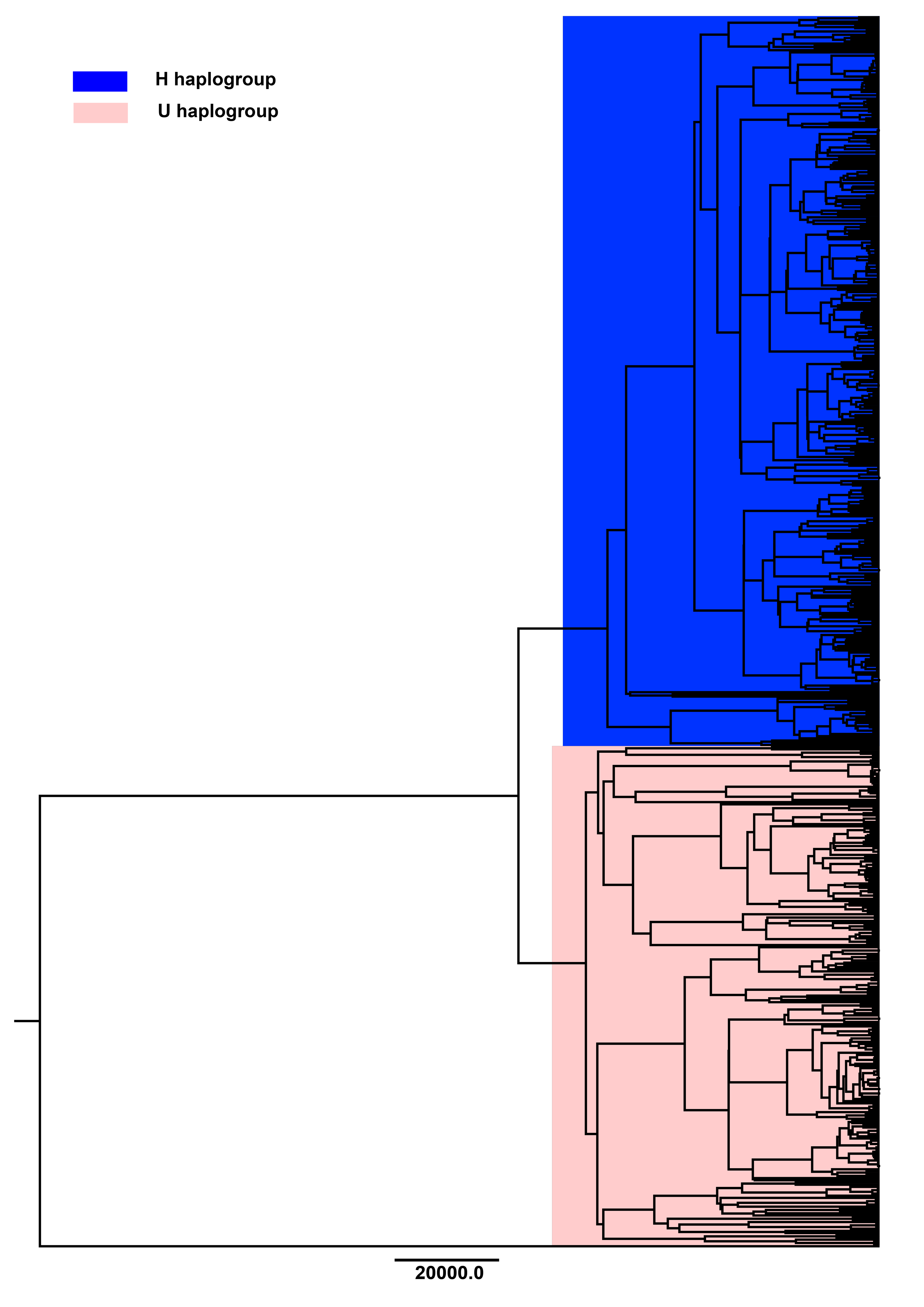

Supplement: Figure S7 — Phylogenetic tree of mtDNAs from dataset 1. The phylogeny was estimated with a Bayesian approach under a GTR+I+R model using 332 present-day European mtDNA sequences of haplogroup H and 228 sequences from haplogroup U. The outgroup is a African mtDNA sequence. (TIF) [file pone.0032473.s007.tif]

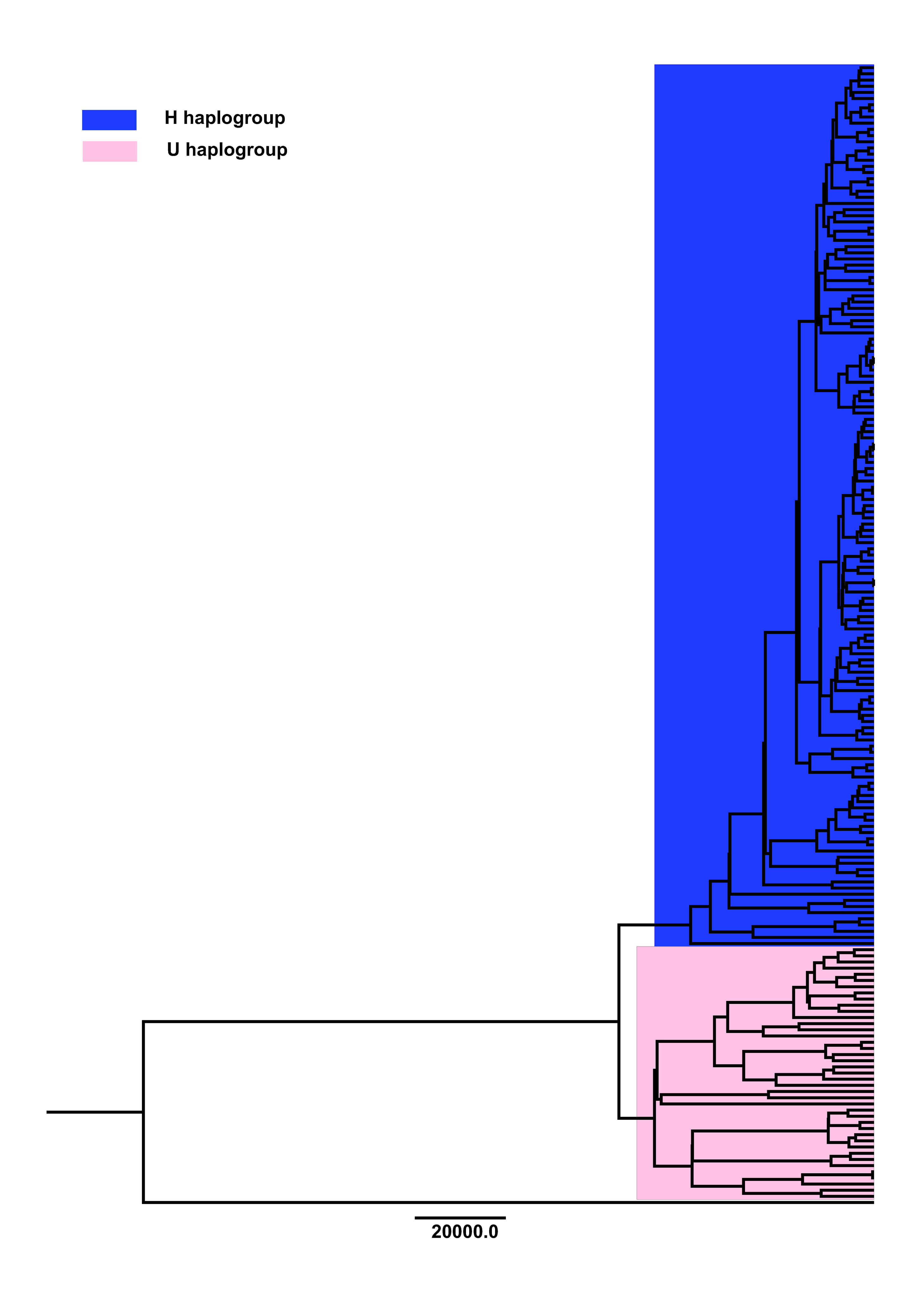

Supplement: Figure S8 — Phylogenetic tree of mtDNAs from dataset 2. The phylogeny was estimated with a Bayesian approach under a GTR+I+R model using 144 present-day European mtDNA sequences of haplogroup H and 41 sequences from haplogroup U. The outgroup is the African mtDNA sequence. (TIF) [file pone.0032473.s008.tif]

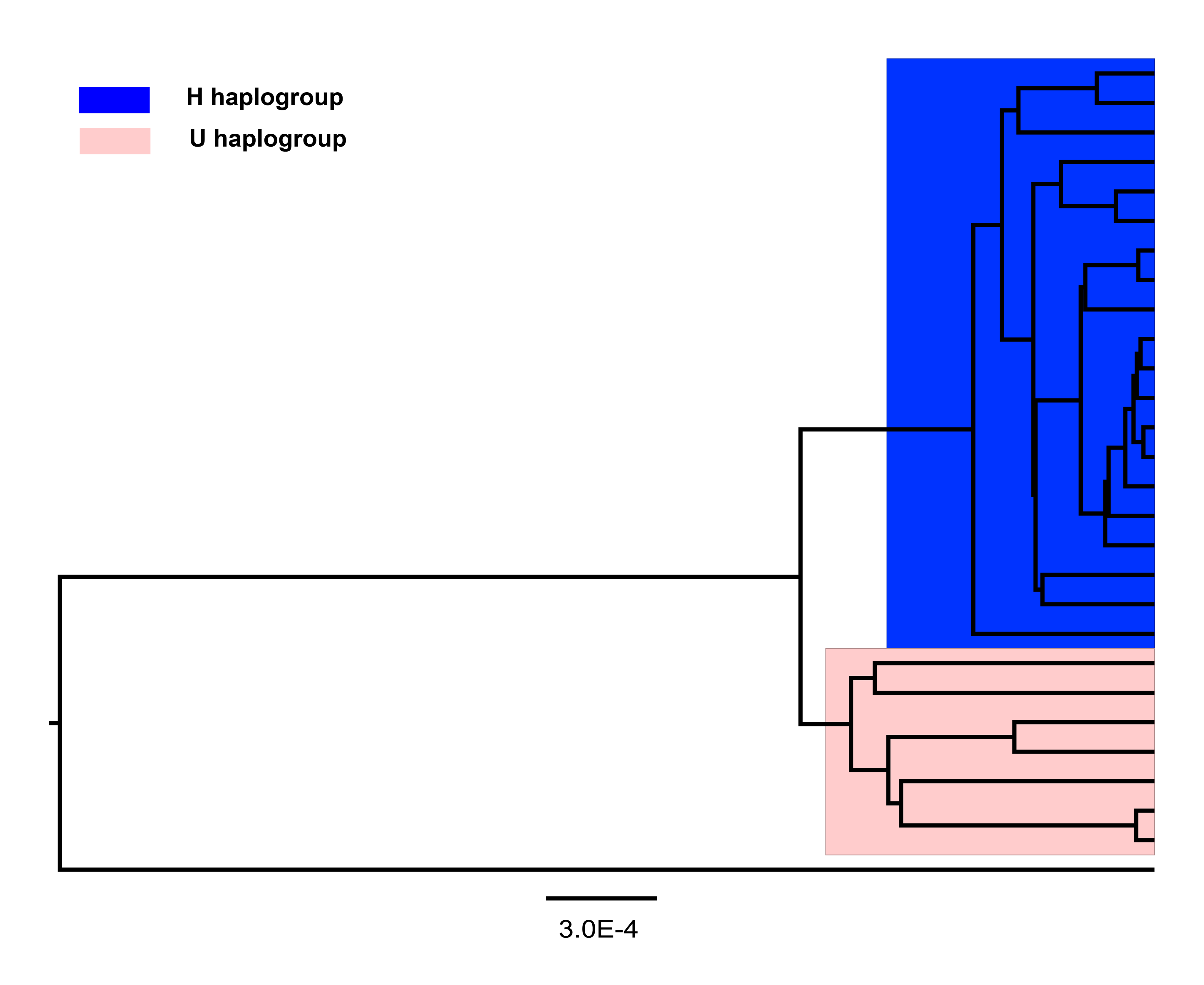

Supplement: Figure S9 — Phylogenetic tree of mtDNAs of dataset 3. The phylogeny was estimated with a Bayesian approach under a GTR+I+R model using 20 present-day Croatian mtDNA sequences of haplogroup H and 7 sequences from haplogroup U. The outgroup is the African mtDNA sequence. (TIF) [file pone.0032473.s009.tif]
