# Supplementary material for: Complete Mitochondrial Genomes Reveal Neolithic Expansion into Europe
Source: PLoS One. 2012 Mar 13;7(3):e32473. doi: 10.1371/journal.pone.0032473 (PMC3302788; doi:10.1371/journal.pone.0032473)
Supplement: Supporting Method S1 — Supporting sequence information of Croatians. (DOC) [file pone.0032473.s010.doc]

# Supporting Sequence Information of Croatia

**DNA extraction and enrichment**

DNA was extracted from blood obtained from 50 villagers sampled in the Northeast and Northwest of Croatia (Figure S1). After shearing the DNA fragments to around 200 base pairs (bp), Illumina libraries were produced from 23µl of sonicated DNA extract .

After library preparation all samples were PCR amplified and the amplified libraries were used for targeted mtDNA capture using the primer extension capture (PEC) protocol. For this protocol 574 biotinylated primers that carry a 5’-biotin are used to bind mtDNA fragments on streptavidin coated beads . Sample libraries were combined in equimolar concentrations in three separate pools and enriched in parallel.

**Illumina/Solexa sequencing**

Library pools were PCR amplified after PEC enrichment with 7 cycles with 60°C annealing temperature in 100µl reactions containing 50µl Phusion^TM^ High-Fidelity Master Mix, 500nM of the p5 and p7 Illumina primers . Subsequently quantification of the amplified product was performed on an Agilent 2100 Bioanalyzer DNA 1000 chip. The enriched and reamplified libraries were pooled in equimolar concentrations and sequenced on the *Illumina Genome Analyzer IIx* platform (FC-104-400x v4 sequencing chemistry and PE-203-4001 cluster generation kit v4) using 2x76 cycles and two seven base pair index reads . The second index was read after the reverse read by repeating the instrument instructions of the first index read. An indexed control PhiX 174 library (index 5’- TTGCCGC-3') was spiked into each lane yielding a fraction of 2-3% control reads in all lanes of the run. Sequencing data was analyzed starting from QSEQ sequence files and CIF intensity files from the Illumina Genome Analyzer RTA 1.6 software. Raw reads were aligned to the φX174 reference sequence to obtain a training data set for the base caller Ibis . Paired-end read sequences called by Ibis 1.1.5 were subjected to a read fusion process (, including removal of adapter sequences and adaptor dimers) by requiring at least an 11nt overlap between the two reads. In the overlapping sequence, quality scores were combined and the base with the highest base quality score was called. Only sequences merged in this way were used for further analysis.

**MtDNA assemblies of the Croatian samples**

In total 27 samples from Northeastern Croatia and 23 from Northwestern Croatia (Table S1) were sequenced and resulted in a total of 12,553,272 merged reads. Using a custom iterative mapping assembler (MIA, Green et al. unpublished, available from the authors upon request), in total 381,833 (3%) of the merged sequences could be aligned to the revised Cambridge Reference Sequence (NC_012920.1, ). These were then used to call sample consensus sequences for positions covered at least twice (Table S1). Comparing GC content and coverage for every sequence position (Figure S2), the distributions are not highly correlated. However, the regions of low coverage and high coverage are similar between samples. Based on mitochondrial coverage, 38 samples whose mtDNA overlapping regions span 13,687 bases were used for analysis.

1. Meyer M, Kircher M (2010) Illumina sequencing library preparation for highly multiplexed target capture and sequencing. Cold Spring Harb Protoc 2010: pdb prot5448.

2. Briggs AW, Good JM, Green RE, Krause J, Maricic T, et al. (2009) Targeted Retrieval and Analysis of Five Neandertal mtDNA Genomes. Science 325: 318-321.

3. Kircher M, Sawyer S, Meyer M Double indexing overcomes inaccuracies in multiplex sequencing on the Illumina platform. Nucleic Acids Research (accepted).

4. Kircher M, Stenzel U, Kelso J (2009) Improved base calling for the Illumina Genome Analyzer using machine learning strategies. Genome Biol 10: R83.

5. Kircher M, Heyn P, Kelso J (2011) Addressing challenges in the production and analysis of Illumina sequencing data. BMC Genomics 12: 382.

6. Andrews RM, Kubacka I, Chinnery PF, Lightowlers RN, Turnbull DM, et al. (1999) Reanalysis and revision of the Cambridge reference sequence for human mitochondrial DNA. Nat Genet 23: 147.
